# Giant duodenal diverticulum masquerading as a sealed perforation

**DOI:** 10.1259/bjrcr.20210196

**Published:** 2022-02-04

**Authors:** Faraaz Khan, Madhurima R Chetan, Horace D'Costa

**Affiliations:** 1 University of Oxford Medical School, Oxford, United Kingdom; 2 Oxford University Hospitals NHS Trust, Oxford, United Kingdom

## Abstract

Giant duodenal diverticula are large outpouchings involving all layers of the duodenal wall. Whilst often an incidental finding, giant duodenal diverticula can present with diverticulitis or biliary obstruction. We report a case of a giant duodenal diverticulum that was initially misdiagnosed as a localised duodenal perforation on CT. Additional ultrasound and fluoroscopic imaging demonstrated the final diagnosis of acute cholecystitis. The clinical course of this patient highlights the challenge of recognising a giant duodenal diverticulum and the limitations of solely relying on CT in the context of an acute abdominal presentation.

## Introduction

Giant duodenal diverticula are a relatively common incidental finding in patients who undergo abdominal imaging. A literature search returns several case reports of giant duodenal diverticula but to our knowledge, these are mostly in the context of raising awareness or drawing attention to the relevant complications, such as diverticulitis, haemorrhage or biliary obstruction (Lemmel syndrome). This case highlights the complexity of making a diagnosis of a giant duodenal diverticulum and the value of imaging modalities other than CT, including ultrasound and fluoroscopy, in diagnosing abdominal pathology. Furthermore, although knowledge of the referrer’s clinical suspicion is necessary when reporting a CT study, it may lead to bias when it comes to subtle imaging features in complex cases. Incorrectly identifying a giant duodenal diverticulum as a duodenal perforation in this patient would have had significant implications for their management and prognostication.

## Case report

**Figure 1.**
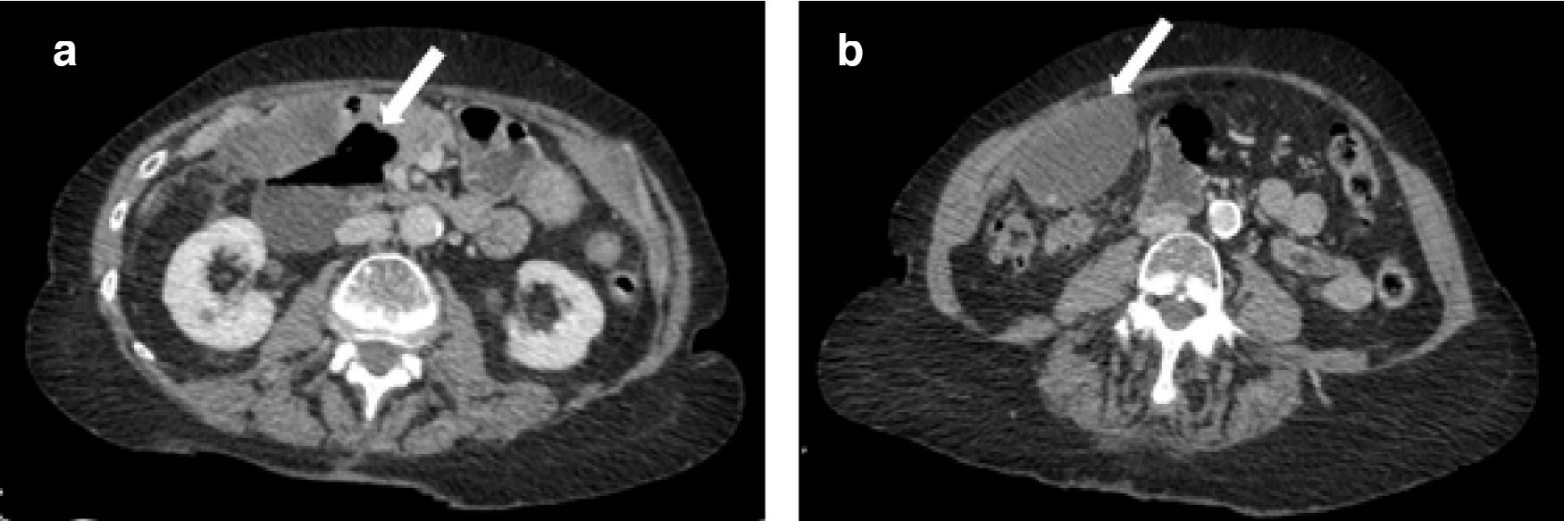
A: CT on the day of admission demonstrates a large 7.8 × 9.3 x 4.6 cm fluid- and gas-containing focus adjacent to the duodenum (arrowed) with associated fat stranding. B: The adjacent gallbladder is distended and mildly thick-walled (arrowed) and contains a calcified gallstone.

An 82-year-old female presented to hospital at night with a 2 day history of worsening epigastric pain that radiated to the right upper and lower quadrants. Her relevant blood results included a white cell count of 16.2 × 10^9^  l^−1^ and a C-reactive protein of 163 mg l^−1^. On clinical examination, the patient had epigastric tenderness with localised guarding. Duodenal perforation was suspected and a CT of the abdomen and pelvis was requested. CT demonstrated a peripherally enhancing large fluid- and gas-containing focus adjacent to the duodenum (Figure 1). It was also noted that fat stranding and a small volume of free fluid were present adjacent to this on the right side of the abdomen. Given the clinical context, this was interpreted by the on-call radiology registrar as a collection related to localised duodenal perforation. Intravenous antibiotics were promptly started and considerations of interventional radiology drainage or total parenteral nutrition and surgical management were made.

**Figure 2.**
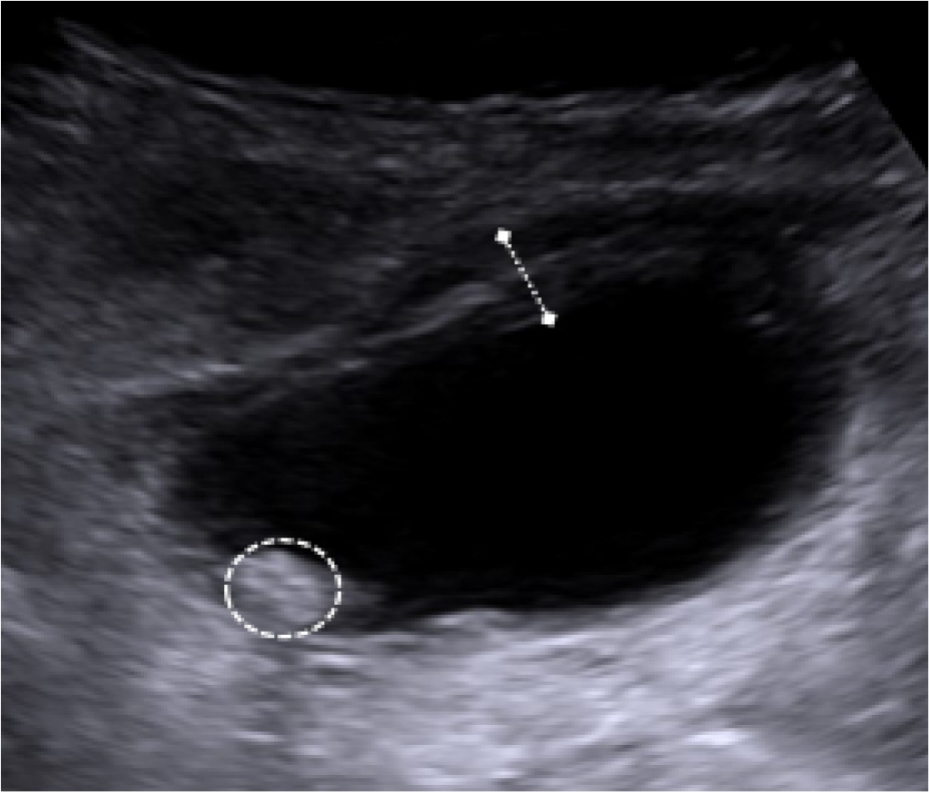
Ultrasound of the gallbladder obtained 1 day post-admission shows a thick-walled gallbladder containing a gallstone (circled).

**Figure 3.**
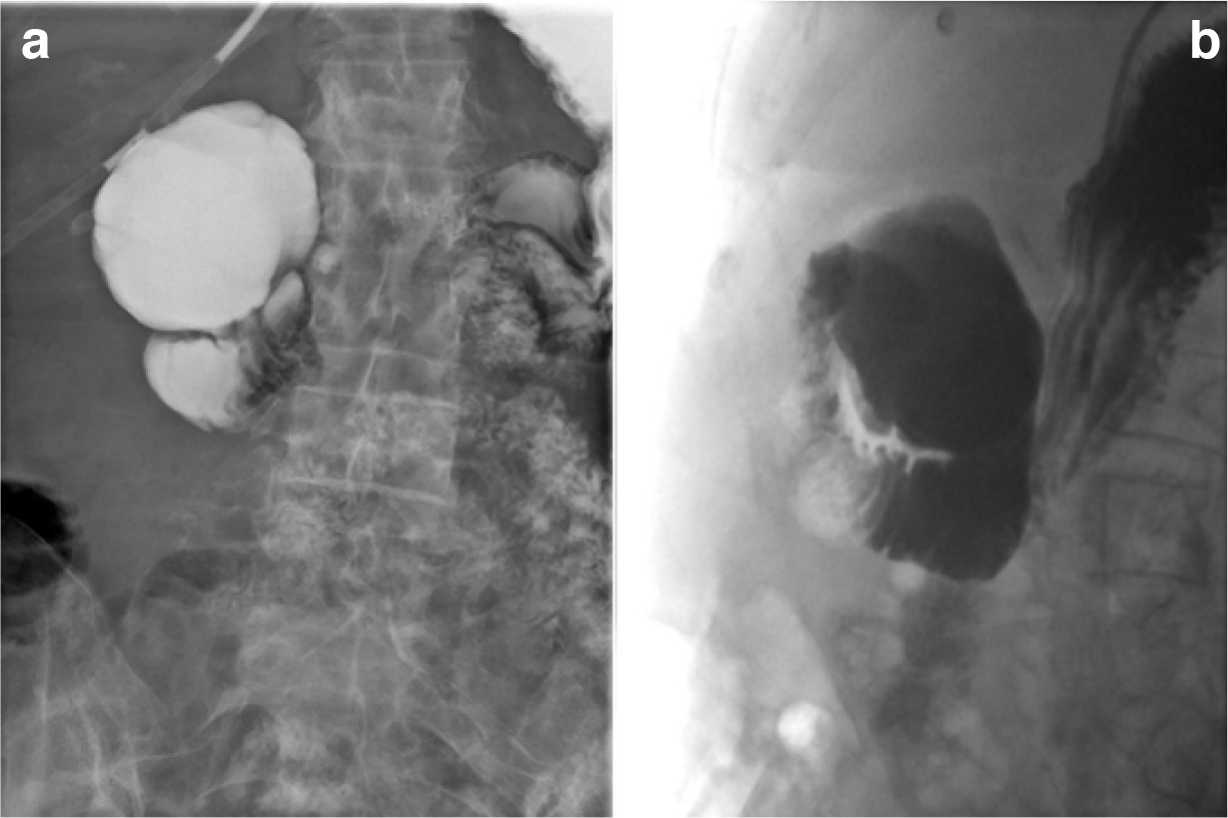
Fluoroscopic images from the water-soluble contrast meal performed 2 days post-admission shows a giant outpouching arising from the duodenum, without extraluminal leak of contrast.

Upon consultant radiologist review in the morning, it was noted that the gallbladder was distended and mildly thick-walled with gallstones. The consultant radiologist considered a diagnosis of cholecystitis with an incidental giant duodenal diverticulum, and suggested an abdominal ultrasound and contrast swallow and meal. The ultrasound subsequently confirmed gallbladder wall thickening and cholelithiasis in keeping with acute calculous cholecystitis (Figure 2). The water soluble contrast study demonstrated contrast passing from the duodenum to fill a 5 cm spheroidal outpouching indicating a giant duodenal diverticulum at D1/D2 (Figure 3). Importantly, no extraluminal tracking of contrast into the retroperitoneum or intraperitoneal cavity was identified to indicate perforation.

**Figure 4.**
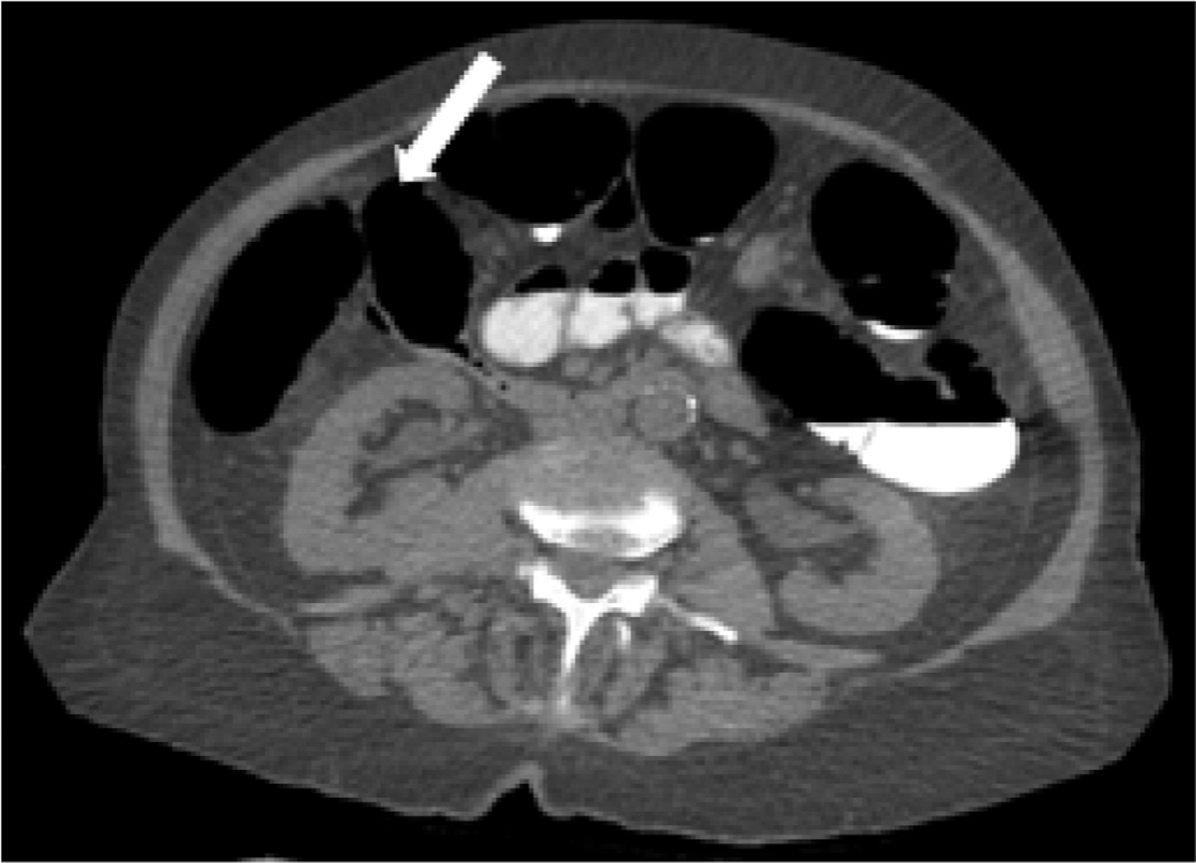
CT virtual colonoscopy study 3 months prior to this admission shows the giant duodenal diverticulum (arrowed) without any surrounding inflammatory stranding.

With a diagnosis of acute cholecystitis and incidental giant duodenal diverticulum, the patient was managed conservatively with intraveous antibiotics, clinically improved, and was discharged. Although no complications of the giant duodenal diverticulum have arisen, the patient had recurrent cholecystitis needing a cholecystostomy later that month and a laparoscopic choelcystectomy 4 months later. On retrospective review of the patient’s available imaging, a giant duodenal diverticulum was present but was not commented on before this admission (Figure 4).

## Discussion

Duodenal diverticula are the second most common site of bowel diverticula, after colonic, and are relatively common with an estimated incidence of 15–22% with the vast majority being asymptomatic and identified incidentally on imaging or post-mortem.^1^ These diverticula are most commonly present near the Ampulla of Vater on the medial side of the bowel wall.^2^ If complications arise then the patient may present with non-specific symptoms such as fever, abdominal pain, weight loss and fatigue. Complications of giant duodenal diverticula include but are not limited to haemorrhage, perforation, fistula formation, bowel obstruction and Lemmel syndrome (diverticulum causing mechanical obstruction of the bile duct and obstructive jaundice).^3–5^ Accordingly, Lemmel syndrome should be considered in the context of abnormal liver functions tests and dilated proximal bile ducts.

Given the clinical presentation, it was necessary for a CT to be performed with the aim of ruling out a suspected duodenal perforation. However, this case highlights the difficulty of maintaining a fully objective approach to reporting CT studies with the knowledge of the patient’s presentation and the clinician’s impressions. Incorporating clinical context into the reporting process can enhance diagnostic accuracy and is prerequisite for a *clinical* radiologist.^6,7^ However, this case highlights that this practice comes with the increased risk of confirmation bias.^8^ It is possible that additional abnormalities related to cholecystitis were initially missed due to attention being preferentially invested in the hypothesised region of interest, in this case, the duodenum. This is compounded by the fact that CT is not the initial modality of choice to diagnose cholecystitis, thus, making it harder to identify these subtle radiological signs. The combination of these cognitive biases and non-ideal modality undoubtedly contributed to the misinterpretation of the fluid and gas collection as perforation.

Notably, it was the use of ‘older’ modalities, namely ultrasound and fluoroscopy, which enabled the correct diagnosis to be reached and the most appropriate patient management plan implemented. The ultrasound was able to identify the classic findings of gallbladder wall thickening and the presence of a calculi, in keeping with cholecystitis. In parallel, fluoroscopy revealed a large outpouching from the duodenum with an abnormal collection of contrast.

A greater awareness of giant duodenal diverticula would be of benefit to ensure patients, particularly those presenting with an acute abdomen, receive appropriate management. For this case, if surgery was carried out for a duodenal perforation then an intraoperative conversion to cholecystectomy would have been necessary. Alternatively, if drainage of the suspected collection was performed then iatrogenic perforation of the diverticulum would have been inevitable, resulting in a significantly worse outcome for the patient. Both scenarios were avoided due to the use of multiple imaging modalities and recognition of a giant duodenal diverticulum. As this diverticulum was identified on retrospective analysis of previous imaging, this case also serves as a reminder that use of previous imaging may aid in determining the key pathology that is driving the patient’s current condition and that which is incidental. Importantly, solely reading previous reports in lieu of analysing previous imaging should be avoided otherwise there is an increased risk of satisfaction of report, thus negating the tangible benefits of previous imaging.^8,9^

The identification of the giant duodenal diverticulum not only had implications on the patient’s initial admission but also future hospital visits if she were to return with abdominal pain. The complications of giant duodenal diverticula will now be more readily considered as a differential.

## Learning points

- Giant duodenal diverticulum is an important relatively common incidental finding to be aware of to ensure it is not misidentified and other abdominal pathologies are recognised
- A multimodality approach to diagnosis is superior to CT alone in the context of this patient’s abdominal presentation.
- Knowledge of the patient’s presentation and likely clinical diagnosis is useful but should not influence the objectivity of a radiological report.
- Radiologists should be aware of and manage the cognitive bias arising from the clinical request and patient notes.
- Reviewing prior imaging can aid the diagnostic process.
